# Seafood Substitutions Obscure Patterns of Mercury Contamination in Patagonian Toothfish (*Dissostichus eleginoides*) or “Chilean Sea Bass”

**DOI:** 10.1371/journal.pone.0104140

**Published:** 2014-08-05

**Authors:** Peter B. Marko, Holly A. Nance, Peter van den Hurk

**Affiliations:** 1 Department of Biology, University of Hawai’i at Mānoa, Honolulu, Hawai’i, United States of America; 2 Indian River State College, Fort Pierce, Florida, United States of America; 3 Department of Biological Sciences, Clemson University, Clemson, South Carolina, United States of America; Stony Brook University, Graduate Program in Public Health, United States of America

## Abstract

Seafood mislabeling distorts the true abundance of fish in the sea, defrauds consumers, and can also cause unwanted exposure to harmful pollutants. By combining genetic data with analyses of total mercury content, we have investigated how species substitutions and fishery-stock substitutions obscure mercury contamination in Patagonian toothfish (*Dissostichus eleginoides)*, also known as “Chilean sea bass”. Patagonian toothfish show wide variation in mercury concentrations such that consumers may be exposed to either acceptable or unacceptable levels of mercury depending on the geographic origins of the fish and the allowable limits of different countries. Most notably, stocks of Patagonian toothfish in Chile accumulate significantly more mercury than stocks closer to the South Pole, including the South Georgia/Shag Rocks stock, a fishery certified by the Marine Stewardship Council (MSC) as sustainably fished. Consistent with the documented geography of mercury contamination, our analysis showed that, on average, retail fish labeled as MSC-certified Patagonian toothfish had only half the mercury of uncertified fish. However, consideration of genetic data that were informative about seafood substitutions revealed a complex pattern of contamination hidden from consumers: *species* substitutions artificially inflated the expected difference in mercury levels between MSC-certified and uncertified fish whereas *fishery stock* substitutions artificially reduced the expected difference in mercury content between MSC-certified and uncertified fish that were actually *D. eleginoides*. Among MSC-certified fish that were actually *D. eleginoides*, several with exogenous mtDNA haplotypes (i.e., not known from the certified fishery) had mercury concentrations on par with uncertified fish from Chile. Overall, our analysis of mercury was consistent with inferences from the genetic data about the geographic origins of the fish, demonstrated the potential negative impact of seafood mislabeling on unwanted mercury exposure for consumers, and showed that fishery-stock substitutions may expose consumers to significantly greater mercury concentrations in retail-acquired fish than species substitutions.

## Introduction

**Figure 1:**
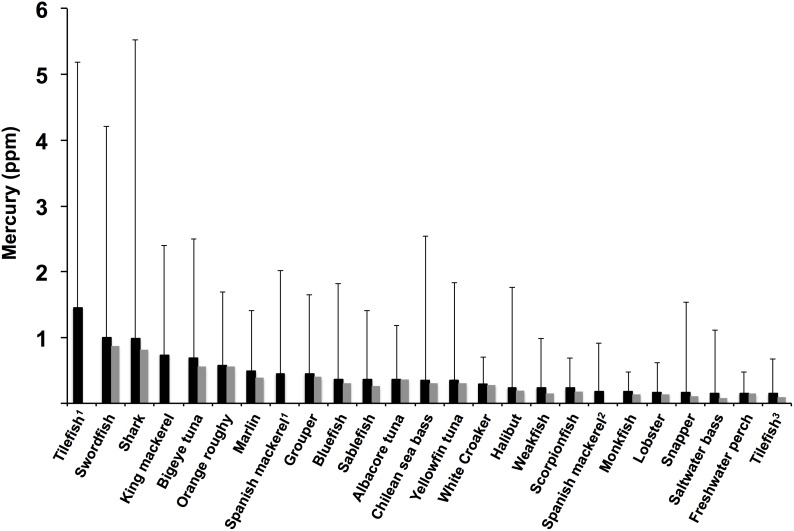
Mercury levels in commercial seafood. Data compiled from US FDA, 1990–2010 (http://www.fda.gov/food/foodborneillnesscontaminants/metals/ucm115644.htm. Accessed 2014 July 14). Dark bars are means, grey are median values. Error bars show the range of measurements. ^1^Gulf of Mexico; ^2^South Atlantic; ^3^Atlantic. Data from canned and fresh albacore were similar and combined.

Despite the many health benefits of eating fish, most commercially harvested fish are contaminated with mercury [1]. The most common form of mercury in fish is methylmercury, a neurotoxin that is especially dangerous to the developing nervous system [2]. Although present in only small quantities in the environment, mercury accumulates in living organisms. Among fish, accumulation of mercury is prevalent but variable (Fig. 1), primarily due to differences in trophic level and body size, such that mercury concentrations tend to be high in larger, longer-lived predatory fish [3]. Therefore, the amount of fish and the particular species of fish consumed are considered the most important factors determining the health risk associated with eating seafood contaminated with mercury. Consequently, the US Food and Drug Administration (FDA) advises pregnant and nursing women, women who may become pregnant, and young children not to eat species that have mean mercury concentrations near 1.0 ppm (shark, swordfish, king mackerel, and tilefish), and not to consume more than 12 ounces per week of other species that have lower mercury levels.

Less widely appreciated is the fact that variation in mercury concentration within species can also be large due to the same causal factors mentioned above [4]–[6]. As a consequence, even though the mean mercury level for many species commonly consumed is only ∼0.3 ppm, the range of mercury concentrations reported from several of these moderately contaminated species often exceeds import limits (Fig. 1). For example, stocks of the southern hemisphere Patagonian toothfish (*Dissostichus eleginoides*), or “Chilean sea bass”, have been shown to have anywhere from ∼0.15 ppm Hg to >1.0 ppm Hg, often well beyond the allowable level of 0.5 ppm for imports into New Zealand, Canada, and Australia, and sometimes greater than the 1.0 ppm limit set by the European Union and the United States [4], [6]–[10].

As with many other species of fish that absorb toxins at a greater rate than they are eliminated, mercury levels in Patagonian toothfish are explained in part by body size: the larger, and presumably the older a fish is, the greater the mercury concentration [7]–[10]. However, two recent studies have shown that mercury accumulation in Patagonian toothfish also has a strong geographic component. Specifically, fish from higher latitudes in the Southern Ocean have lower length-normalized mercury concentrations compared to fish from lower latitudes [9]–[10]. In particular, fish from the South Georgia/Shag Rocks (SGSR) stock have mean mercury levels far below allowable import levels worldwide, with a mean THg of 0.23 ppm (SE = 0.01 ppm) compared to fish from nearby Chile, which have more than three times as much mercury in their tissues (0.73 ppm, SE = 0.10 ppm) [9]. Mercury levels in fish taken from the SGSR stock are of particular interest given that several consumer-education (e.g., Seafood Watch) and seafood sustainability certification programs (e.g., Marine Stewardship Council or MSC) consider fish from SGSR populations as eco-friendly seafood choices, from the perspectives of sustainability and relatively low by-catch. This combination makes fish from certified-sustainable southern ocean stocks a potentially attractive choice for consumers interested in purchasing sustainably harvested seafood with consistently low levels of mercury.

Accurate labeling of seafood is essential to allow consumers to reliably choose sustainable fisheries [11]–[15], but labels may also serve to protect consumers from unhealthy mercury exposure. For example, several common *species* substitutions (i.e., the substitution of less desirable species for more expensive ones) can result in unintended high mercury consumption, such as the substitution of tilefish or king mackerel for grouper [12], [17]–[18]. However, some *fishery-stock* substitutions also have the potential to significantly increase mercury exposure to consumers. For example, Gulf of Mexico tilefish averages 1.45 ppm Hg, but THg in Atlantic tilefish is an order of magnitude lower (Fig. 1). Tuna species from the Atlantic, Pacific and Mediterranean oceans also have significantly different length-normalized mercury concentrations [4].

Here, we report measured mercury concentrations in Patagonian toothfish or “Chilean sea bass” acquired from retail merchants in the USA. As an indicator for species and fishery-stock substitutions, we used genetic data from the same samples that showed 20% of fish labeled as “Chilean sea bass” were not *D. eleginoides* (8% of fish sold as MSC-certified Chilean sea bass were other species whereas 46% of uncertified fish were substitutes), and among those certified fish positively identified with DNA techniques as *D. eleginoides*, 15% had mtDNA haplotypes that were not present in the previously described MSC-certified stock [16]. Our analysis of mercury contamination in the genetically-identified samples shows that the pattern of mercury contamination is consistent with the interpretation of mislabeling based on mtDNA haplotype identities, and demonstrates that in addition to species substitutions, fishery-stock substitutions have the potential to significantly obscure mercury exposure for consumers.

## Methods

### Samples

The tissue samples used in this study were a subset of those used in an analysis of the genetic composition of retail-acquired “Chilean Sea Bass” [16]. All of the samples were purchased “fresh” (i.e., from the thawed seafood counter) from retailers in 10 US states as filets (body wall muscle), which were immediately stored in 90% ethanol to preserve mtDNA. Samples were acquired from retailers selling either MSC-certified Chilean sea bass (and were clearly labeled as such) or from retailers selling fish as Chilean sea bass, but not as MSC-certified. We measured the total mercury (THg) concentration in 25 of the MSC-certified fish and 13 of the non-MSC fish.

### Total mercury concentration

Samples stored in ethanol were freeze-dried in a VirTis lyophilizer for 72 h. Of the dried fish tissue, 70–100 mg weighed samples were digested in sealed Teflon containers with 6 ml nitric acid in a 1 ppm Au background according to EPA method 3052 [19]. Samples were microwaved at 400 W for 25 min, allowed to cool and taken up with water. Samples were then analyzed for total mercury content by ICP-MS according to EPA method 200.8 [20]. For every 5 samples a blank was run, and two reference samples of oyster tissue were included in the sample series. All quality control samples fell within acceptable ranges, and the limit of detection was 0.096 µg/g. To make our dry-weight measurements of THg comparable to wet-weight concentrations in the literature, we converted our THg measurements into wet-weight concentrations by dividing by the widely accepted conversion factor of 3.6 [21].

### Statistical Analysis

Because some comparisons involved unequal sample sizes, we first tested for unequal variances with an F-test and then used t-tests (either for equal or unequal variances) to examine the data for differences in THg among groups of samples. P-values were calculated as one-tailed given the expectation that MSC-certified fish (which should have originated from SGSR fishery) should have lower levels of THg than fish that originated from non-certified fisheries at lower latitudes in the southern hemisphere [9]. Because we performed three t-tests on overlapping subsets of the data, we considered P-values significant if <0.05/3 or 0.0167.

## Results

**Figure 2:**
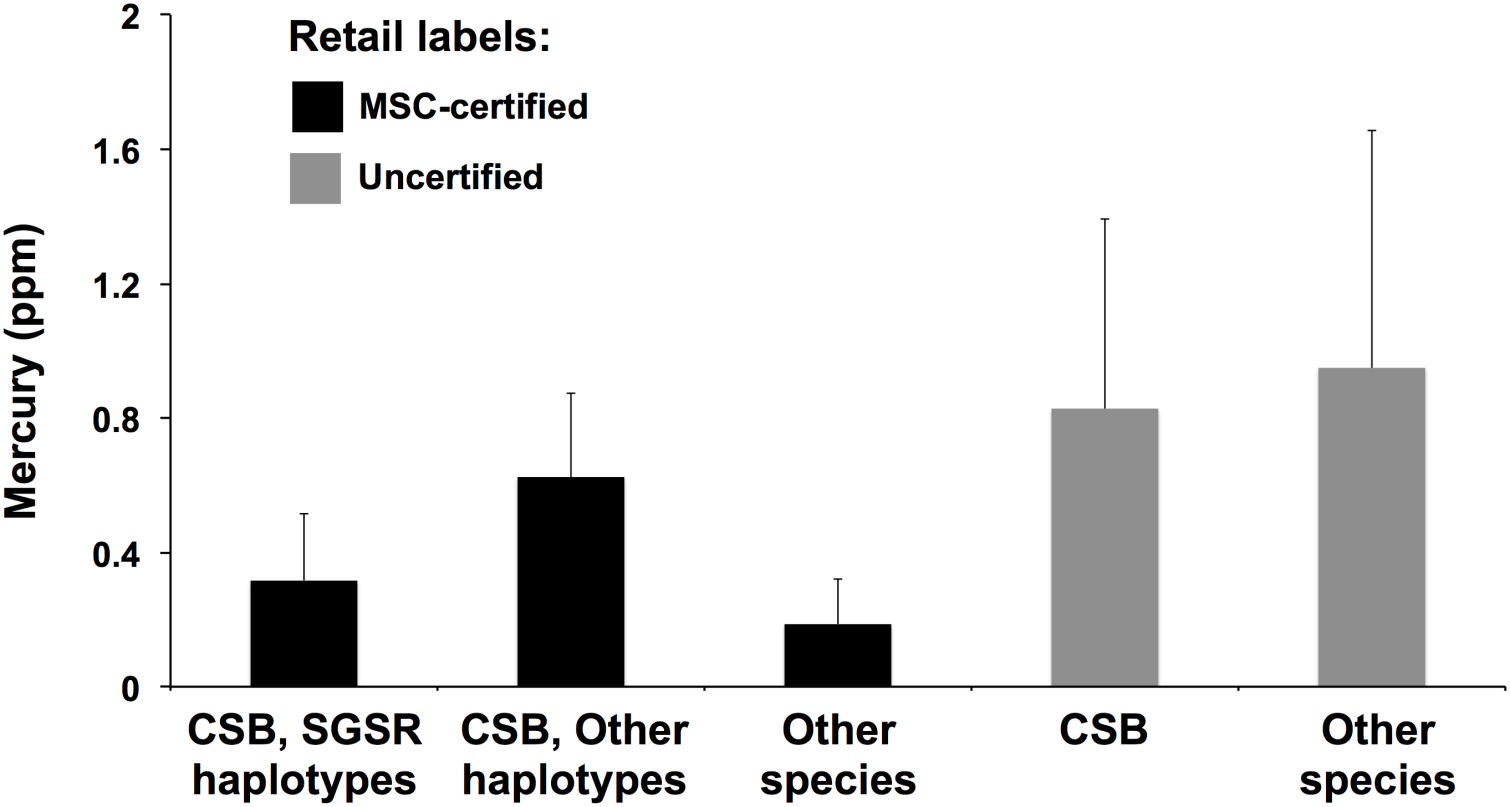
Mercury concentrations among samples of retailed acquired Patagonian toothfish (*Dissostichus eleginoides*) or Chilean sea bass (CSB) in this study. Error bars are standard deviations.

Measurements of THg for the retail-acquired Chilean sea bass in our study varied widely among samples, from a low of 0.07 ppm to a high of 1.9 ppm. Overall, fish labeled as MSC-certified Chilean sea bass had less than half the THg of uncertified fish (Table 1, Fig. 2), a highly significant result (t-test for unequal variances: t = −2.97, one-tailed p = 0.0050). However, among only those fish that were genetically verified as *D. eleginoides*, the difference in THg between MSC-certified and uncertified fish was only marginally significant at the 0.05 level and not significantly different at the 0.0167 level (t-test for unequal variances: t = −2.15, one-tailed p = 0.0320).

**Table 1:** Total mercury (THg) concentration of fish sold as Chilean sea bass (CSB) with and without MSC labels, indicating their origin from the certified South Georgia (SG) fishery based on genetic analyses.

Samples	N	THg (ppm)	SD
***MSC-labeled fish***			
i) All fish	25	0.35	0.24
ii) CSB only	22	0.42	0.24
Known SG haplotypes	17	0.31	0.20
Unknown SG haplotypes	5	0.63	0.25
Excluding Heard Island haplotype	4	0.72	0.16
***Non-MSC fish***			
i) All fish	13	0.89	0.60
ii) CSB only	8	0.80	0.56

**Figure 3:**
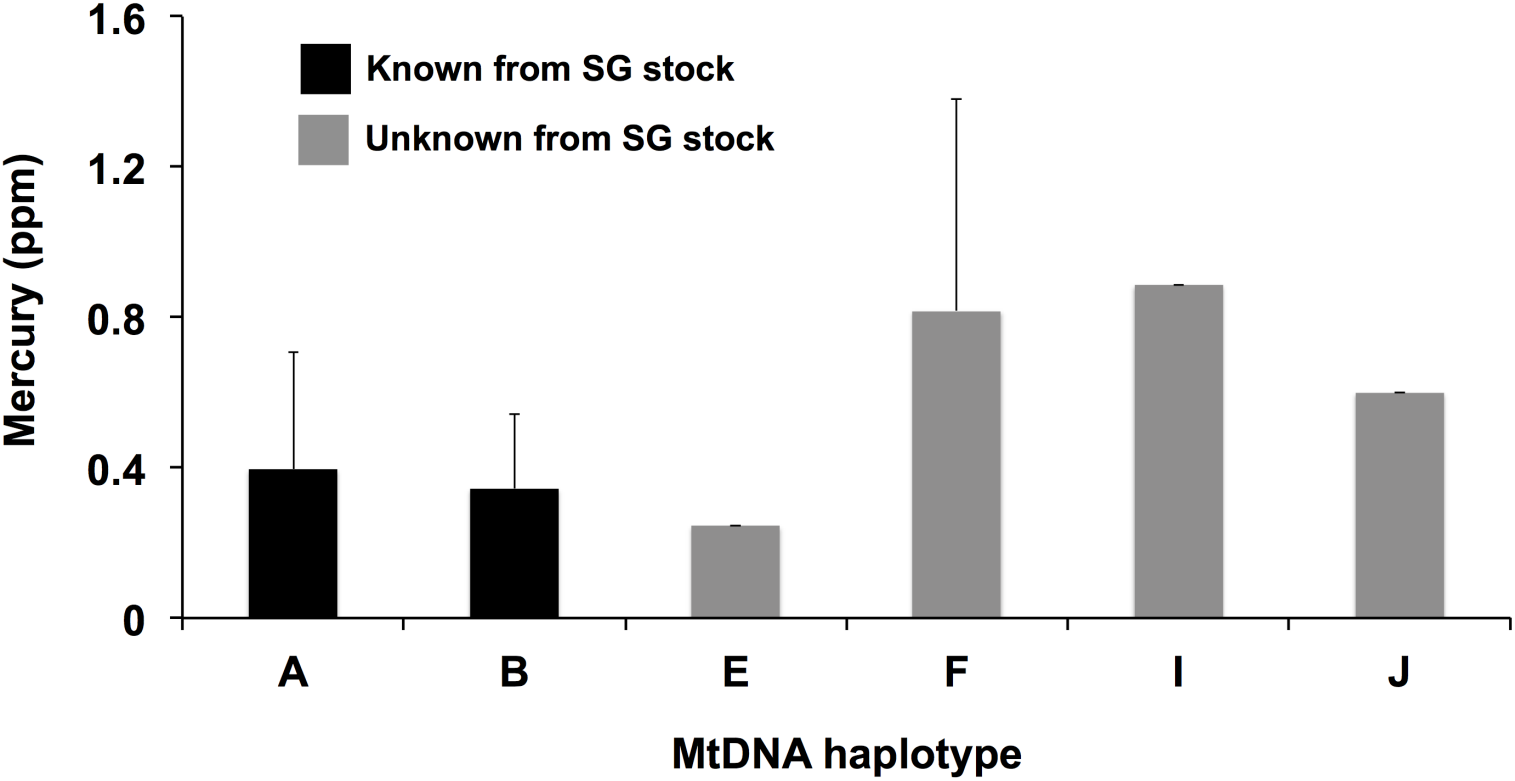
Mercury concentrations among mitochondrial DNA haplotypes from MSC-certified retail samples of Patagonian toothfish (*Dissostichus eleginoides*) or Chilean sea bass. Error bars are standard deviations.

For MSC-certified fish that were actually *D. eleginoides*, we found an uneven distribution of THg contamination: those with mtDNA haplotypes not known from the MSC-certified fishery (haplotypes E, F, I, & J) had twice as much mercury (0.63 ppm) as *D. eleginoides* with haplotypes known from the South Georgia stock (0.31 ppm) (Fig. 3; t-test for equal variances: t = −2.87, one-tailed p = 0.0047). The single fish with haplotype E was unusual among fish with haplotypes unknown from the certified fishery in that it had relatively low THg (Fig. 3).

## Discussion

Our study of retail acquired Patagonian toothfish or “Chilean sea bass” demonstrated that both species substitutions and fishery-stock substitutions obscure a complex pattern of mercury contamination. First, although fish labeled as MSC-certified “Chilean sea bass” appeared to have significantly lower total mercury than uncertified fish, consideration of genetically detected *species* substitutions (i.e., substitutions of other species for *D. eleginoides*) revealed the unexpected result that among fish that were actually *D. eleginoides*, the difference in mercury content between MSC-certified and uncertified fish was small. However, this unexpected result is explained in part by the finding that the species that were substituted for MSC-certified fish had relatively low mercury (mean THg = 0.14 ppm) whereas species substituted for uncertified fish most commonly had very high mercury (mean THg = 0.95 ppm). Together, these species substitutions combined to artificially inflate the difference in mercury levels between all MSC-certified and uncertified fish.

In contrast, *fishery-stock* substitutions (substitutions of *D. eleginoides* from uncertified stocks for *D. eleginoides* from certified stocks) also likely contributed to the apparently small difference in mercury content between MSC-certified and uncertified fish that were verified genetically as *D. eleginoides*: several MSC-certified fish that were genetically verified as *D. eleginoides* had relatively high mercury, twice the concentration of most certified fish in our study (Fig. 3). Given that all of these toothfish with unexpectedly high mercury also had mtDNA haplotypes unknown from the certified SGSR fishery, their high mercury content is consistent with the idea that they did not originate from the certified fishery [16]. Two of these four fish with exogenous haplotypes had haplotype F, the dominant haplotype on the Patagonian shelf [22], where fish are known to accumulate higher mercury levels than compared to SGSR fish [9]. One fish labeled as MSC-certified (but which did not possess a haplotype known from the certified SGSR fishery) had a haplotype known only from Heard Island [16]. As with SGSR, Heard Island lies south of the Antarctic Polar Front, an oceanographic barrier that likely restricts gene flow in *D. eleginoides* [22] and might protect the Southern Ocean from mercury contamination [9]. Consistent with a high-latitude origin, this putative Heard Island fish had correspondingly low mercury (0.24 ppm, Fig. 1), outside the 99% confidence interval (0.26–1.18 ppm) for the four other fish with haplotypes not known from the certified fishery. Although the Heard Island longline toothfish fishery is now MSC-certified, it was not certified at the time the samples in our study were purchased [16].

The potential for species substitutions to cause unintended mercury exposure for consumers has been described before, but our study of Patagonian toothfish is novel in that it shows that fishery-stock substitutions, which are more difficult to detect than species substitutions, can expose consumers to increased mercury concentrations in retail-acquired fish. For example, although species substitutions result in a decrease in mean mercury concentrations of ∼7% (for MSC-certified fish) and an increase of ∼11% (for uncertified fish), fishery-stock substitutions (among MSC-labeled fish) resulted in an increase in mean mercury of ∼100% (Table 1). Because Patagonian toothfish from Chile often exceed the 1.0 ppm FDA mercury import threshold (and vastly exceed limits for Canada, New Zealand, and Australia) [9], our results suggest that in addition to species’ identities, the geographic origins of fish may be worthy of greater consideration in seafood consumption studies.

A potential confounding factor in our results may be that we were not able to correct mercury concentrations with body size of the sampled fish, because the samples were obtained from fish filets that were sold at seafood counters in retail stores. Therefore, an alternative explanation for the unusually high THg of some fish labeled as having originated from the SGSR stock is that they were simply very large fish from SGSR. However, given that the MSC-labeled fish with exogenous mtDNA haplotypes (excluding the fish with haplotype E, known only from Heard Island) had an average mercury concentration of 0.72 ppm (Table 1), those fish must have had average lengths of approximately 160 cm if actually from SGSR (see Fig. 2 from 9), more than 45 cm longer than any fish obtained in earlier studies of mercury in SGSR fish [9], a fishery with a modal catch length of only 75 cm [23]. Furthermore, these MSC-labeled fish with unusually high mercury levels also had mtDNA haplotypes not known from the SGSR stock (two of which are the most common haplotype in Chilean waters) and mercury levels expected for Chilean fish ranging in size from 75 to 100 cm, suggesting the mislabeling hypothesis is a better explanation for MSC-certified fish with unexpectedly high mercury levels.

Although all of our samples were stored in ethanol, our mercury measurements were consistent with previous measurements from Patagonian toothfish. For example, we found a mean mercury level of 0.31 ppm for toothfish with haplotypes known from the SGSR stock whereas a mean mercury level of 0.23 ppm was previously reported for frozen fish (i.e., not preserved in ethanol) sampled from the same fishery [9]. The relative amounts of mercury between fish from north and south of the Antarctic Polar Front in the south Atlantic/Southern Ocean were also similar between our study and earlier work: frozen samples from Chile had 3.1 times the mercury content of fish from SGSR [9], similar to a factor of 2.7 in our study in a comparison of uncertified but genetically identified *D. eleginoides* (most of which were labeled as having originated from Chile) to MSC-certified Chilean sea bass that had mtDNA haplotypes known from the certified fishery. Therefore, our results are consistent with substantially increased mercury exposure (twice as much) from fish that did not originate further south [9]–[10].

## Conclusions

Our study found considerable retail variation in mercury levels in “Chilean sea bass” that is related to the likely geographic origins of the fish, but also indicated that species substitutions and fishery-stock substitutions may obscure apparent levels of mercury among fish labeled as both MSC-certified and uncertified Patagonian toothfish. Although on average, MSC-certified fish is a healthier option with respect to mercury contamination than compared to uncertified fish, our study showed that fishery-stock substitutions, can result in a larger proportional increase in mercury consumption than species substitutions for consumers, and that variation in mercury contamination among fishery stocks may be considered in future seafood consumption guidelines.
